# Indications for an EEG in a Child with ADHD

**DOI:** 10.15844/pedneurbriefs-29-5-6

**Published:** 2015-05

**Authors:** J. Gordon Millichap, John J. Millichap

**Affiliations:** 1 Division of Neurology, Ann & Robert H. Lurie Children's Hospital of Chicago, Chicago, IL; 2 Departments of Pediatrics and Neurology, Northwestern University Feinberg School of Medicine, Chicago, IL

**Keywords:** Attention-Deficit/Hyperactivity Disorder, Digit Span, Rolandic Spikes

## Abstract

Investigators at Departments of Child Neurology, Neuroscience, Biostatistics, Marmara University, Istanbul, Turkey studied the parameters for prediction of epileptiform abnormalities in the EEG of 148 children diagnosed with ADHD, according to DSM-IV criteria, aged between 6 and 13 years (mean 8.76 +/- 1.26; 25.7% female). Wake and sleep EEGs lasting about one hour were obtained in 89.2% patients and a WISC-R in 100%.

Investigators at Departments of Child Neurology, Neuroscience, Biostatistics, Marmara University, Istanbul, Turkey studied the parameters for prediction of epileptiform abnormalities in the EEG of 148 children diagnosed with ADHD, according to DSM-IV criteria, aged between 6 and 13 years (mean 8.76 +/- 1.26; 25.7% female). Wake and sleep EEGs lasting about one hour were obtained in 89.2% patients and a WISC-R in 100%. The coexistence of speech sound disorder and higher Digit Span test performance predicted the occurrence of epileptiform discharges in the EEG. The prevalence of epileptiform abnormalities was 26.4%; they were frequently localized in the frontal (41%) and centrotemporal (28.2%) regions. Speech sound disorder co-occurrence (64%) with rolandic spikes suggests that the pathophysiology of epileptiform abnormalities in ADHD might be genetically determined. [1]

COMMENTARY. The utility of the EEG in a child presenting with ADHD is a frequent and sometimes hotly debated subject. The American Academy of Pediatrics (AAP), in their 2000 clinical practice guideline for diagnosis of ADHD [2], found no reliable differences between the EEG in children with ADHD and normal controls. Higher rates of abnormal EEGs in some studies lack consistency, and “the current literature is not supportive of routine EEG in the diagnosis of ADHD.” Two references are provided, one showing that the EEG of children diagnosed with ADHD in psychiatry clinics in Japan, China, and Korea are significantly different from those of normal and deviant behavior groups, showing more delta and theta and fewer alpha waves [3]. A second reference to a study using quantitative EEG showed differences in the ADHD, ADD, and control groups [4]. ADHD is associated with increased fronto-central theta and theta/beta ratio compared to controls [5].

Whereas the studies in the 1990s, in agreement with the AAP guideline, are not supportive of the routine use of the EEG in diagnosis of ADHD, subsequent research regarding the utility of the EEG in the management of patients with ADHD shows that an increased susceptibility to epilepsy is sufficiently strong to warrant investigation. Of 624 records (94.5% sleep-deprived) of nonepileptic children evaluated for ADHD in our clinic and laboratory, 163 (26.8%) were abnormal, 42.9% only focal spikes, chiefly central, frontal, and temporal [6]. Seven studies, 2000-2011, found epileptiform abnormalities in an average of 25.1% of children with ADHD. The prevalence of EEG seizure discharges does not warrant a trial of AED but caution in the use of stimulants is advised. An ADHD child who exhibits episodic altered awareness or a family history of epilepsy should be considered for an EEG. The present report adds the coexistence of speech sound disorder and higher digit span to the indications for an EEG in ADHD. The 2000 AAP guidelines may require revision based on more current literature [5–7].
